# Expression and prognostic role of STAT5a across cancer types

**DOI:** 10.1042/BSR20230612

**Published:** 2023-08-02

**Authors:** Christine Maninang, Jinghong Li, Willis X. Li

**Affiliations:** Department of Medicine, University of California San Diego, La Jolla, CA, U.S.A.

**Keywords:** breast cancers, meta analysis, STAT5a

## Abstract

Studies examining the role of signal transducer and activator of transcription 5 (STAT5) in various cancers have produced controversial results. To address this controversy, we examined the prognostic role of STAT5a in cancer patients across multiple cancers. Transcription levels of STAT5a between tumors and normal tissues, obtained from public databases, were analyzed for statistical differences using Cox regression analysis with the outcome as overall survival and covariate of interest as high STAT5a expression. Meta-analysis was then conducted to summarize the hazard ratio estimate from the Cox regression analyses. We found that STAT5a was significantly under-expressed in breast, lung, and ovarian cancers, while STAT5a was significantly overexpressed in lymphoid neoplasm diffuse large B-cell lymphoma, glioblastoma, and glioma. High STAT5a expression was significantly associated with favorable survival in bladder cancer (lnHR = −0.8689 [−1.4087, −0.3292], *P*-value = 0.0016), breast cancer (lnHR = −0.7805 [−1.1394, −0.4215], *P*-value < 0.0001) and lung cancer (lnHR = −0.3255 [−0.6427, −0.0083], *P*-value = 0.0443). After adjusting for clinicopathological factors, high STAT5a expression remained significantly associated with favorable survival in breast cancer (lnHR = −0.6091 [−1.0810, −0.1372], *P*-value = 0.0114). These results suggest that higher STAT5a expression is associated with favorable overall survival in breast cancer, and therefore might have protective effects, and that STAT5a expression could be a potential prognostic biomarker, especially in breast cancer. However, the prognostic role of STAT5a is dependent on cancer type.

## Introduction

Cancer is a complex and devastating disease. Despite new advancements in research, the disease caused nearly 10 million deaths in 2020, remaining one of the major causes of death globally [1,2]. The development of resistance to standard therapies such as chemotherapy and radiation treatment has become one of the concerning challenges in treating cancer. Therefore, developing new therapeutic approaches and diagnostics is urgent. In recent years, targeted therapy showed some success, demonstrating a need to investigate possible new signaling pathways contributing to cancer and biomarkers to create new therapeutic strategies and tools assisting clinical decisions, thus improving treatments for cancer patients.

Signal transducer and activator of transcription (STAT) proteins have emerged as potential therapeutic targets of interest due to their roles in cancer development and progression [3,4]. Among the seven mammalian STAT proteins, STAT5a is crucial in mammary gland development. It has been demonstrated that STAT5a activation, by phosphorylation, promotes alveolar cell proliferation and differentiation during lactation and mammary epithelial cell survival [5,6]. In addition, STAT5 is crucial in immune functions as it plays a vital role in regulating the generation and fate of T cells. For instance, STAT5 activation has been shown to limit the generation of T helper cells via interleukin-2 signaling [7,8].

Much of the published evidence has shown the oncogenic role of STAT5, particularly its involvement in promoting tumor survival, growth, metastasis, and resistance to cancer treatments in various cancer types. Studies looking at the effect of STAT5 activity in prostate cancer indicated that JAK2 inhibitor or dominant-negative STAT5 inhibits tumor growth and promotes apoptosis [9,10]. Consistent with these findings, a study on clinical outcomes has found that STAT5 activation correlates with higher histological grade, early recurrence, and shorter progression-free survival [11]. Similarly, it has been reported that, in colorectal cancer, phosphorylated or activated STAT5 is associated with shorter overall survival [12]. For hematological cancers, a study demonstrated that activated STAT5 promotes cell growth by upregulating cyclin D2 [13]. Another study showed that inhibition of STAT5 decreases anti-apoptotic proteins while increasing pro-apoptotic proteins [14]. In non-small cell lung cancer (NSCLC) subtypes, STAT5 is overexpressed in the cytoplasm and nucleus of cancer cells [15]. In addition, inhibiting STAT5 activation reduces cell growth and promotes apoptosis in NSCLC cells [16].

However, some studies reported contradictory findings. Studies have shown that STAT5 has a tumor-suppressive function in liver cancer. It has been demonstrated that loss of STAT5 expression results in cancer development and liver fibrosis due to increased STAT3 activation [17]. STAT5 in liver cancer seems to counteract the STAT3 signaling pathway. Further research in liver cancer cells has identified STAT5 target genes, which include Nox4, a reactive oxygen species (ROS) enzyme, and pro-apoptotic proteins p53 up-regulated modulator of apoptosis (PUMA) and Bcl-2-interacting mediator of cell death (Bim), and STAT5 up-regulates the genes encoding these proteins [18]. For breast cancer, studies demonstrated that overexpressed or activated STAT5 promotes tumor formation and growth and slows post-lactational apoptosis [5,19]. However, other studies reported that activated STAT5 inhibits cell proliferation, survival, invasion, metastasis, and angiogenesis [20,21]. In addition, studies have found that STAT5 expression is associated with increased overall survival and better response to endocrine therapy in estrogen receptor (ER)-positive breast cancer, and that activated STAT5 is associated with better breast cancer-specific and disease-free survival in lymph node-negative breast cancer [22,23]. Like in liver cancer, STAT5 also seems to counteract the oncogenic effects of STAT3 as part of the tumor-suppressive role of STAT5 in breast cancer, as suggested by a report that STAT5 and STAT3 activation have opposing effects, and that co-activation of STAT5 and STAT3 in breast cancer cells decreases cell proliferation and sensitizes cells to chemotherapeutic drugs paclitaxel and vinorelbine, in contrast with STAT3 activation alone [24]. The study suggests that STAT5 activation is likely dominant over STAT3. In addition, a study has found that unphosphorylated STAT5a exhibits a tumor suppressor function through binding with heterochromatin protein 1α (HP1α), leading to the stabilization of heterochromatin in colorectal cancer cells [25]. Moreover, the study has shown that the expression of unphosphorylated STAT5a inhibits tumor growth in mouse xenographs, and that down-regulation of STAT5 or HP1α expression in prostate cancer patients is associated with poor disease-free survival [25].

The prognostic value of STAT5 expression in cancers remains unclear. Studies examining the relationship between STAT5 expression and survival outcomes in cancer patients have produced mixed results [26–29]. In this study, we conducted a systematic analysis by pooling and extracting gene expression datasets to examine the gene expression pattern and the prognostic value of STAT5a across all cancer types.

## Methods

### GEPIA database analysis

Gene Expression Profiling Interactive Analysis 2 (GEPIA2) (http://gepia.cancer-pku.cn/) is an interactive database tool consisting of RNA-seq data from tumor and normal tissue samples from The Cancer Genome Atlas (TCGA) and Genotype-Tissue Expression (GTEx) databases [30]. Analysis was performed to identify STAT5a transcription levels in various cancers. Differential expression analysis to compare STAT5a transcription levels of tumor tissues was compared with normal tissue transcription levels. Gene expression was expressed onto box plots, and a one-way ANOVA test was performed to test differences in STAT5a expression between tumor and normal tissues. The *P*-value threshold was set at 0.05.

### Prognoscan database analysis

**Figure 1:**
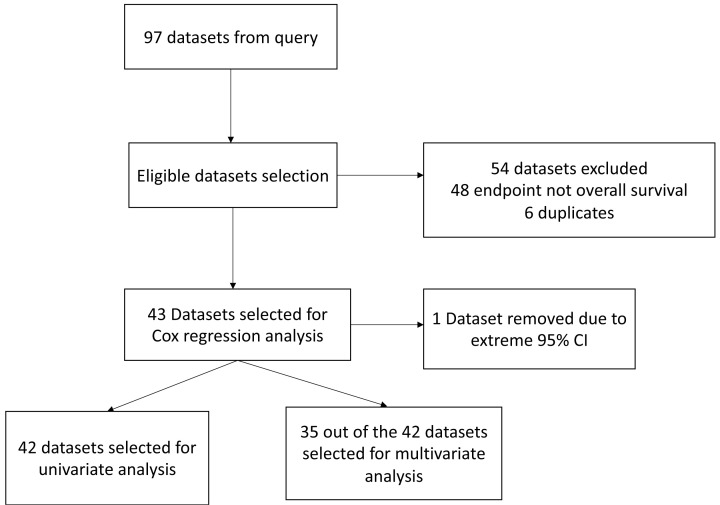
Flow diagram of dataset selection The flow diagram shows the dataset selection process. Datasets were chosen based on the overall survival endpoint. In cases where duplicate datasets with different probes existed, the dataset with the smallest log-rank test* P*-value was selected. This process yielded 42 datasets for univariate Cox regression analysis. Out of these, 35 datasets were further chosen for multivariate Cox regression analysis based on the availability of individual clinical data.

Prognoscan (http://dna00.bio.kyutech.ac.jp/PrognoScan/) is an online platform used to assess potential tumor biomarkers and therapeutic targets from cancer microarray datasets extracted from databases such as Gene Expression Omnibus (GEO), ArrayExpress, and laboratory websites [31]. The database uses the minimum *P*-value approach to determine the optimal cut point for gene expression values to group patients into either high- or low-expression groups. We used Prognoscan to evaluate the prognostic value of STAT5a expression in cancer patients. The term ‘STAT5A’ was entered into the database to obtain datasets. Datasets with the survival endpoint of overall survival were selected. Duplicate datasets with different probes were settled by selecting the dataset with the smallest log-rank test *P*-value. The search resulted in 42 datasets for univariate Cox regression analysis, and 35 of those datasets were selected for multivariate Cox regression analysis based on available individual clinical data (Figure 1 and Supplementary Table S1). We determined the high and low expression of STAT5a based on the minimum *P*-value approach and the Kaplan–Meier plots provided by the Prognoscan database.

### cBioPortal database analysis

Gene alterations of the STAT5a gene were analyzed using the cBioPortal database (http://www.cbioportal.org) [32,33]. The database contains sample data from more than 200 TCGA and non-TCGA studies combined. The parameters for the query included mutations, structural variants, and copy numbers. A bar graph was made using the database features to look at gene alterations frequency and types of STAT5a across cancer types. The minimum number of total cases was set at 100 samples.

### Tumorscape database analysis

We utilized the Tumorscape database (http://www.broadinstitute.org/tumorscape/) [34] to analyze copy number alterations of the STAT5a gene. The Broad Institute of MIT and Havard developed the database and contained more than 3,000 samples. The gene-centric analysis examined the amplifications and deletions of STAT5a across human cancers. The portal creates a results table that includes whether the gene queried is at a peak region of alteration, the *q*-value that indicates the significance of the copy number alterations affecting the gene, the number of genes in peak, and overall, focal, and high-level frequency of each gene alteration type.

### Statistical analysis

Kaplan–Meier curves from microarray datasets were examined for survival differences between STAT5a expression groups. The log-rank test was used to test the survival differences between expression groups. Individual data from datasets were collected from the Prognoscan database to perform Cox proportional hazard analysis. Overall survival was selected as the outcome, and STAT5a expression (high or low) was selected as the main predictor which was treated as a categorical variable. Low expression was set as the reference. A univariate analysis was performed to obtain the hazard ratios (HRs) and 95% confidence intervals (CIs) for the relationship between high STAT5a expression and overall survival. Further, a multivariate Cox proportional hazard analysis for datasets with sufficient clinical annotations. As shown in Supplementary Table S2, covariates with complete data were added to the analysis to obtain the adjusted HRs and CIs for high STAT5a expression. Supplementary Table S3 summarizes how each covariate was treated in the multivariate analysis for each dataset. Assumptions were checked using graphical Schoenfeld tests for both univariate and multivariate analyses. Assumptions did not hold up for all datasets. Therefore, HRs were interpreted as the average HR over time. Next, we conducted a meta-analysis to pool unadjusted and adjusted HRs and CIs using the generic inverse variance method. One dataset was not included in the meta-analysis due to its extremely large 95% CI. Both random-effects and fixed-effect models were performed. Cochran’s *Q-*test and *I*^2^ index were performed to test heterogeneity between datasets. *Q*-test *P*-value < 0.05 and *I*^2^ > 50% was considered significant heterogeneity. Publication bias was assessed using a funnel plot and Egger’s test. Subgroup analysis was done to investigate the relationship between STAT5a expression and overall survival in each cancer type. The significance threshold for all tests was set at *P*-value < 0.05. Cox regression analysis and meta-analysis were conducted in R software (ver. 4.1.3) using the ‘survival’ package and ‘meta’ package respectively.

## Results

### Transcription expression of STAT5a across cancer types

**Figure 2:**
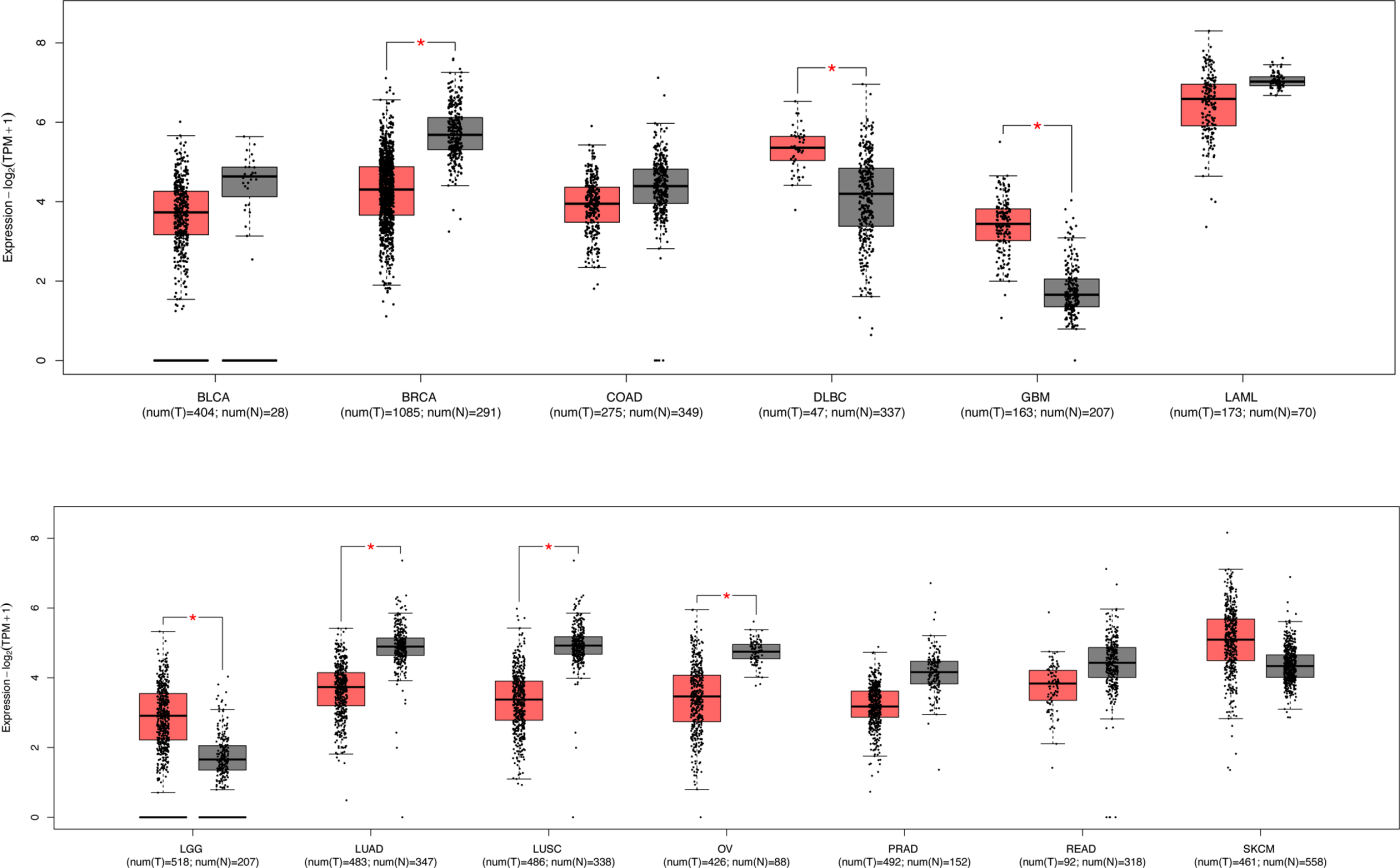
Transcriptional levels of STAT5a in tumor versus normal tissue STAT5a mRNA expression levels in the GEPIA2 database in cancer versus normal tissues are shown. Red = tumor Black = normal, TPM = transcripts per million, log_2_(TPM+1) = log transformation of expression data. BLCA, bladder urothelial carcinoma; BRCA, breast invasive carcinoma; COAD, colon adenocarcinoma; DLBC, lymphoid neoplasm diffuse large B-cell lymphoma; GBM, glioblastoma multiforme; LAML, acute myeloid leukemia; LGG, brain lower grade glioma; LUAD, lung adenocarcinoma; LUSC, lung squamous cell carcinoma; OV, ovarian serous cystadenocarcinoma; PRAD, prostate adenocarcinoma; READ, rectum adenocarcinoma; SKCM, skin cutaneous melanoma.

To investigate the role of STAT5a in cancers, we compared transcription levels of STAT5a in tumor tissues to normal tissues. Using the GEPIA2 database, we found that STAT5a mRNA expression has significantly lower expression in breast, lung, and ovarian cancers, whereas STAT5a mRNA has significant overexpression in lymphoid neoplasm diffuse large B-cell lymphoma, glioblastoma, and glioma compared with normal tissue (Figure 2).

### The prognostic value of STAT5a expression from Prognoscan database

**Figure 3:**
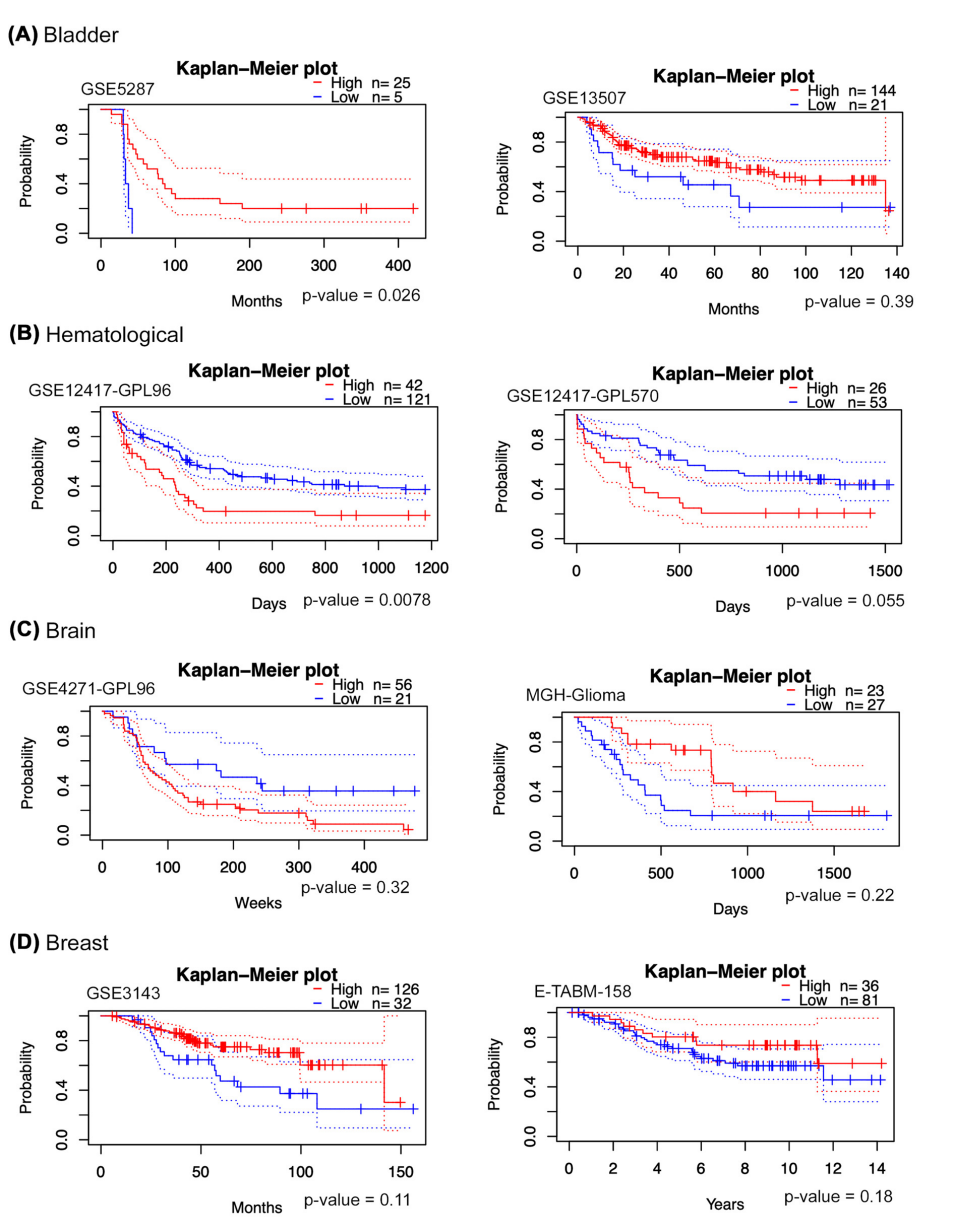

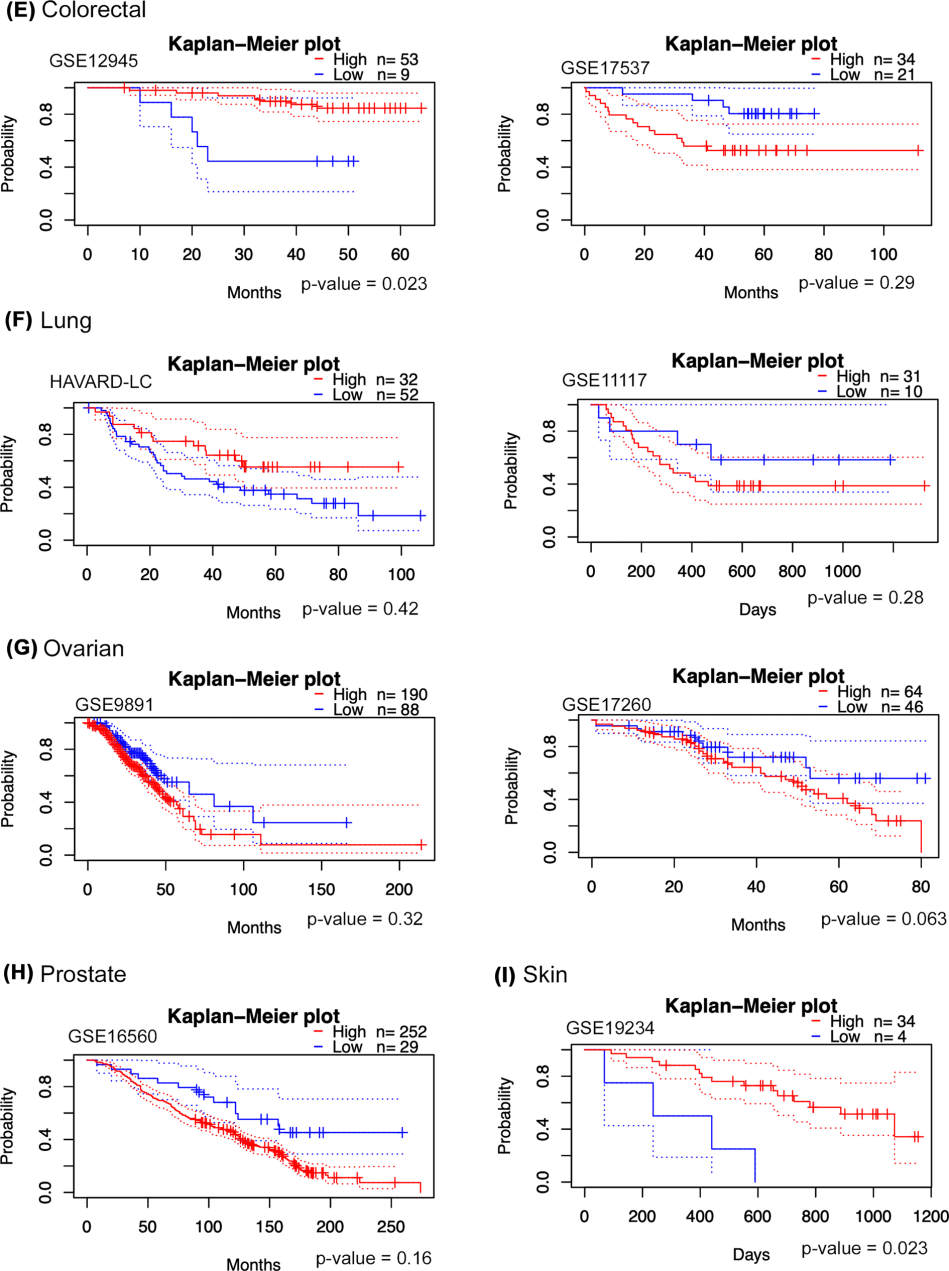
Overall survival between STAT5a high versus low expression in cancer patients Kaplan–Meier plots for overall survival between STAT5a high-expressing cancer patients and low-expressing cancer patients of different cancer types (as indicated A-I) are shown using data from the Prognoscan analysis platform. Note that bladder, breast, and skin cancer patients with high STAT5a expression had favorable overall survival compared to patients with low STAT5a expression.

Using the Kaplan–Meier plots from the Prognoscan analysis platform, we compared the overall survival between STAT5a high-expressing cancer patients and low-expressing cancer patients. We found that bladder, breast, and skin cancer patients with high STAT5a expression had favorable overall survival compared to patients with low STAT5a expression (Figure 3). In contrast, STAT5a low-expressing patients had more favorable overall survival than high-expressing patients in hematological, ovarian, and prostate cancer patients (Figure 3). However, it is important to note that some datasets for hematological and ovarian cancers display opposite findings. The association of high STAT5a expression to overall survival appeared unclear in brain, lung, and colorectal cancer.

We conducted univariate and multivariate Cox regression analysis to investigate the association between high STAT5a expression and overall survival with a low-expression group set as the reference group. HRs, CIs, and Cox *P*-values are summarized in Supplementary Table S4. For the univariate analysis, high STAT5a expression was significantly associated with poor overall survival in prostate cancer (lnHR = 0.6987 [0.1553;1.242], *P*-value = 0.0117) (Supplementary Table S4). In melanoma, high STAT5a expression was significantly associated with favorable overall survival (lnHR = −1.788 [−2.975, −0.6004], *P*-value = 0.00317) (Supplementary Table S4). For the multivariate analysis, high STAT5a expression was significantly associated with poor overall survival in prostate cancer (lnHR = 0.736 [0.1906, 1.281], *P*-value = 0.00818) (Supplementary Table S4).

**Figure 4:**
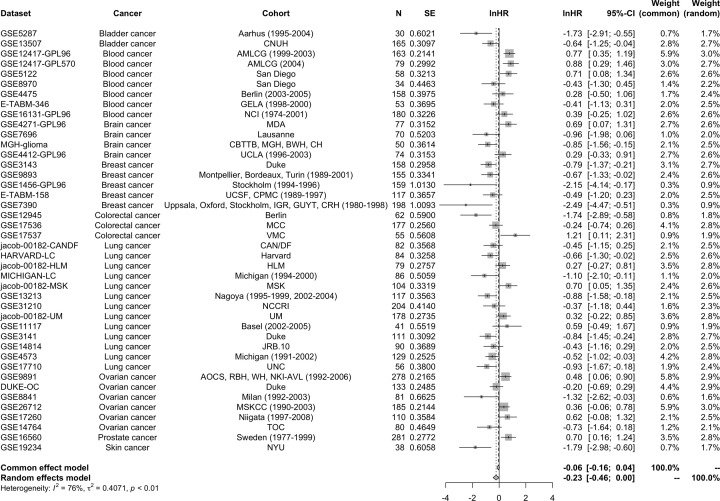
Forest plot of STAT5a unadjusted hazard ratios Overall effect: lnHR = −0.2293 (−0.4560, −0.0025), *P*-value = 0.0475. Meta-analysis was conducted in all selected datasets using hazard ratio estimates from univariate Cox regression analysis. Heterogeneity between studies and significance of the differences are shown (*I*^2^ = 76%, *P*-value < 0.01).

Meta-analysis was conducted in all selected datasets using hazard ratio estimates from univariate Cox regression analysis from Table S3. There was significant heterogeneity between studies (*I*^2^ = 76%, *P*-value < 0.01); therefore, the random effects model was adopted to estimate pooled HR and 95% CI. The pooled HR and 95% CI of overall survival revealed favorable overall survival for high STAT5a expression (lnHR = −0.2293 [−0.4560, −0.0025], *P*-value = 0.0475) (Figure 4).

**Figure 5:**
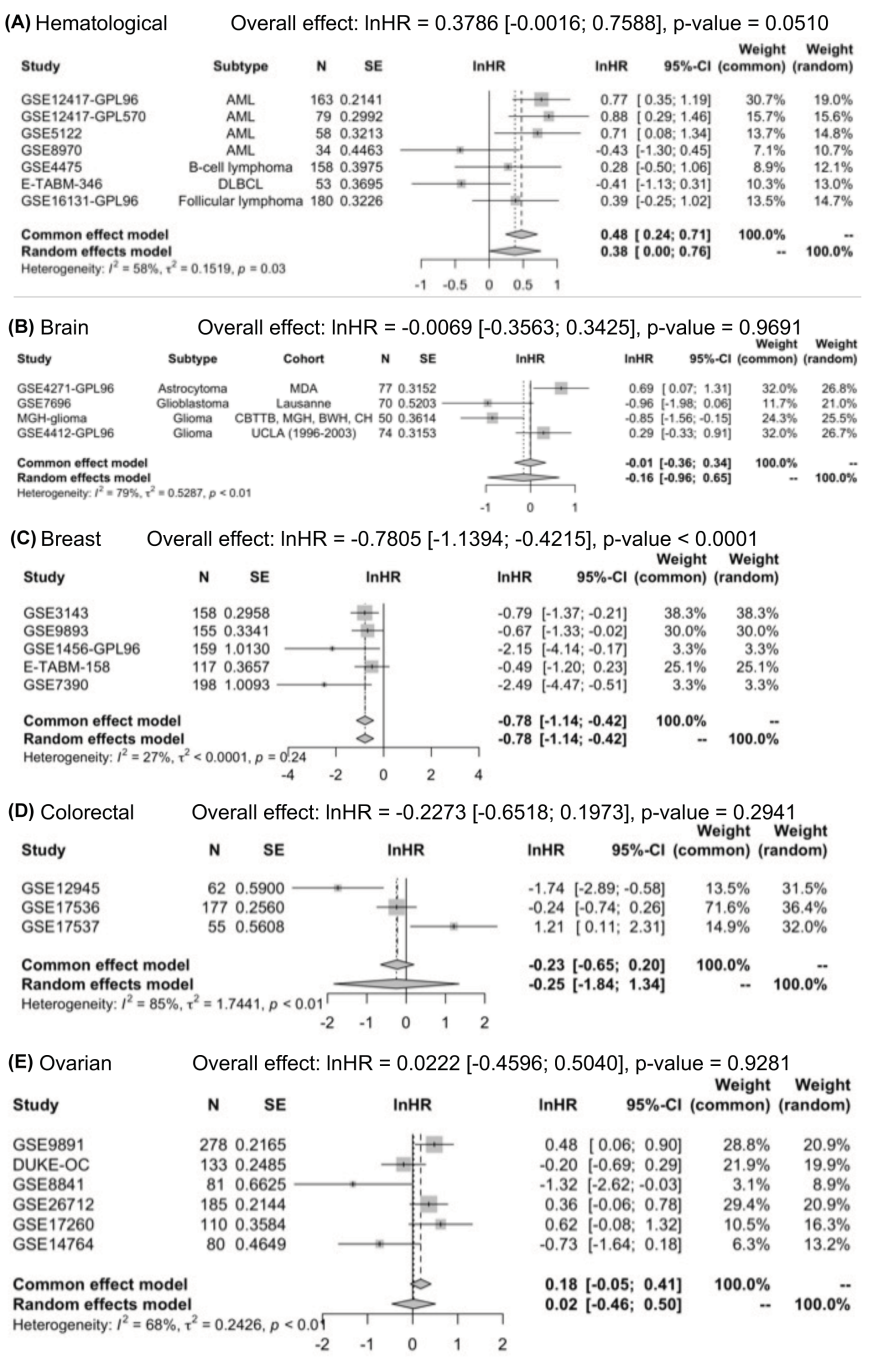

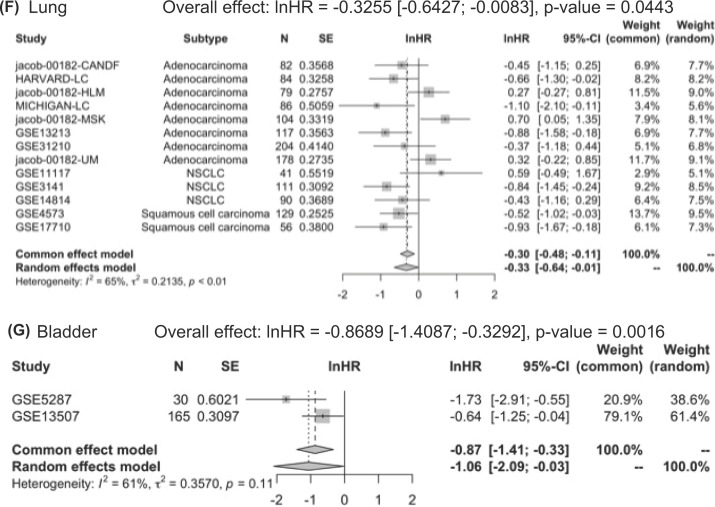
Subgroup analysis of unadjusted hazard ratios by cancer type (as indicated A-G) The overall effect of hazard ratios and significance are indicated.

Due to significant heterogeneity, subgroup analysis and meta-regression analysis were done to explore possible sources of heterogeneity among datasets. Subgroup analysis was conducted to explore the possible differences in high STAT5a expression for overall survival in different cancers using HRs from the univariate analysis. Subgroup analysis showed that STAT5a expression was significantly associated with favorable overall survival in breast (lnHR = −0.7805 [−1.1394, −0.4215], *P*-value < 0.000), lung (lnHR = −0.3255 [−0.6427, −0.0083], *P*-value = 0.0443), and bladder cancer (lnHR = −0.8689 [−1.4087, −0.3292], *P*-value = 0.0016) (Figure 5). There was no significant association between STAT5a and overall survival in hematological, brain, colorectal, and ovarian cancer (Figure 5). Meta-regression analysis indicated that array type and probe ID contribute little to heterogeneity (Array type: *R*^2^ = 6.77%, Probe ID: *R*^2^ = 0.11%).

**Figure 6:**
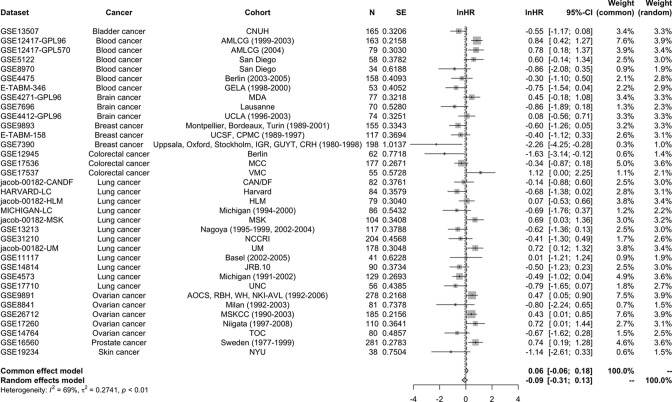
Forest plot of adjusted hazard ratios Overall effect: lnHR = −0.0919 [−0.3116, 0.1279], *P*-value = 0.4126.

A combined analysis of eligible datasets from the multivariate Cox regression analysis was also conducted for eligible datasets from Supplementary Table S3 (Figure 6). There was significant heterogeneity among studies (*I*^2^ = 68%, *P*<0.01). After adjusting for available clinicopathological factors, the combined analysis showed no significant association between STAT5a expression and overall survival in cancer patients. Meta-regression and subgroup analysis was conducted due to the significant heterogeneity between datasets (Figure 6).

**Figure 7:**
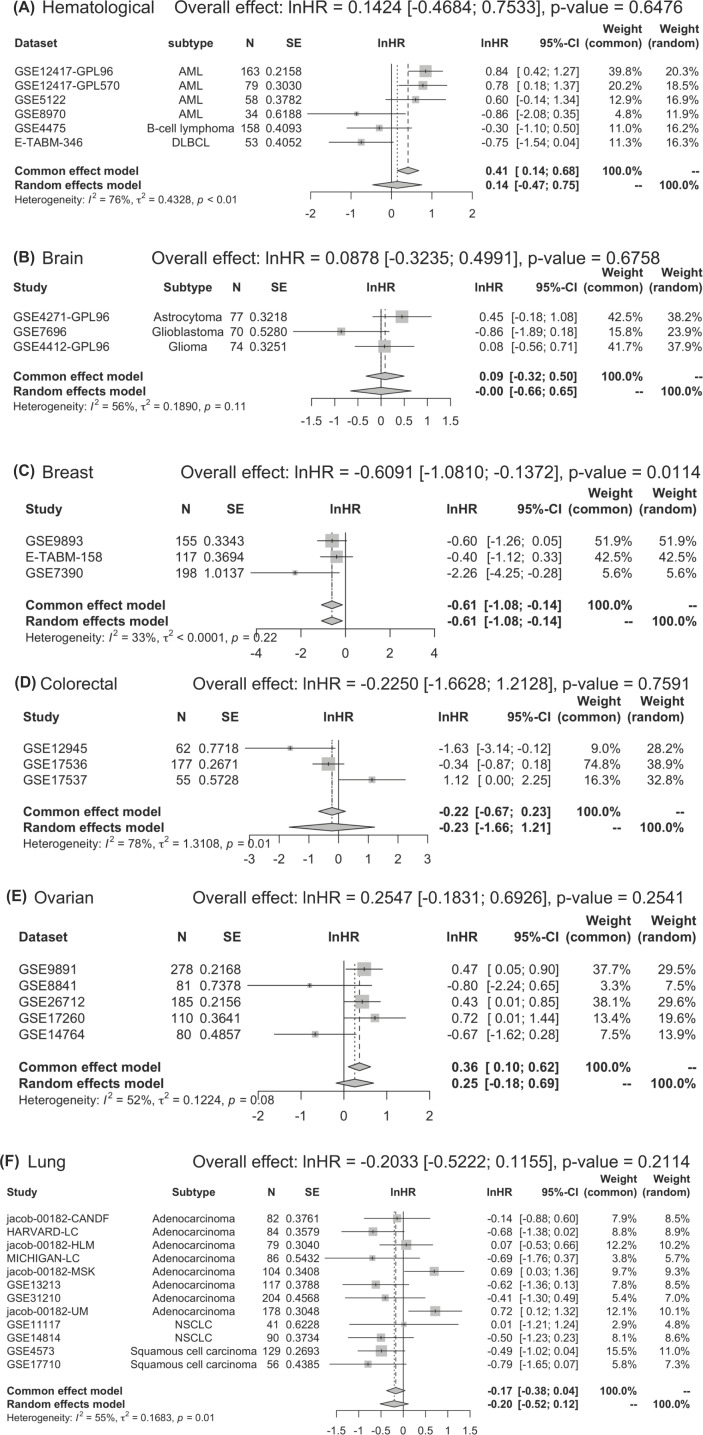
Subgroup analysis of adjusted hazard ratios by cancer type (as indicated A-F) The overall effect of hazard ratios and significance are indicated for each cancer type.

Subgroup analysis of adjusted HRs by cancer types showed that STAT5a expression was significantly associated with favorable overall survival in breast cancer (lnHR = −0.6091 [−1.0810, −0.1372], *P*-value = 0.0114) (Figure 7) aligning with the results from a subgroup analysis of the unadjusted HRs. There was no significant association between STAT5a expression and overall survival in hematological, brain, colorectal, ovarian, and lung cancer. Meta-regression analysis indicated that array type and probe ID did not contribute to heterogeneity among datasets.

### Publication bias

**Figure 8:**
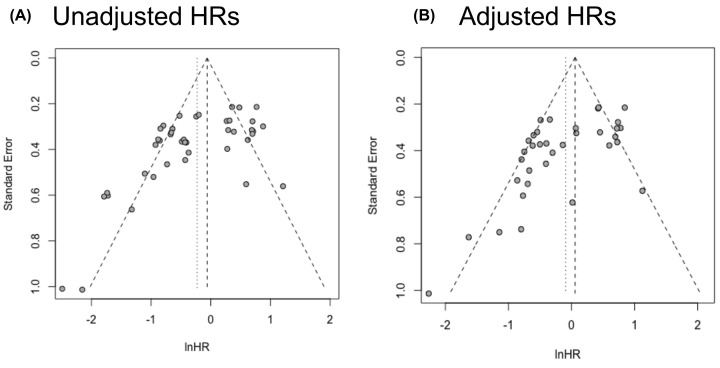
Funnel plots of unadjusted and adjusted hazard ratios Publication bias was analyzed unadjusted (**A**) and adjusted (**B**) hazard ratios.

Finally, we used Egger’s linear regression test and funnel plots to assess potential publication bias in the meta-analysis. The tests indicated statistical evidence of publication bias for both datasets from univariate Cox regression analysis (*P*-value = 0.0003) and multivariate Cox regression analysis (*P*-value = 0.0003) (Figure 8).

### STAT5a gene alteration in different cancer types

Analyzing the gene alteration frequency and mutation types of STAT5a across cancer types using cBioPortal, we found less than 10% alteration frequency across 44 cancer types with a variation in mutation types (Supplementary Figure S1). In addition, we used the Tumorscape database to analyze STAT5a amplifications and deletions across cancer types (Supplementary Tables S5 and S6). These studies demonstrated that deletion of the STAT5a gene was significant across the dataset and in epithelial and breast cancer (Supplementary Table S6), consistent with the expression analyses shown above.

## Discussion

Previous studies have indicated that STAT5a participates in cancer development and progression and could potentially be a therapeutic target in cancers [9–16,19]. However, the role of STAT5a in cancer remains controversial. We conducted analyses to compare STAT5a transcription levels in different cancer types to normal tissues to address this controversy. Using the GEPIA2 analysis platform, we have found that STAT5a was significantly under-expressed in breast, lung, and ovarian cancer compared with normal tissues, consistent with previous reports [26,27,35]. On the other hand, STAT5a was significantly over-expressed in lymphoid neoplasm diffuse large B-cell lymphoma, glioblastoma, and lower-grade glioma. These results indicate that STAT5a may have an oncogenic or tumor-suppressive role dependent on cancer type.

To investigate the potential role of STAT5a as a prognostic marker, we investigated the association between high STAT5a expression and overall survival in cancer patients. The prognostic value of STAT5a gene expression was examined using the Prognoscan platform to compare survival between high STAT5a expressing and low STAT5a expressing patients and then performing a meta-analysis. It is important to note that we only focused on STAT5a expression, not STAT5a activation, which may or may not be correlated to each other. While there was no significant association between hematological, brain, and ovarian cancers in the meta-analysis, high STAT5a expression was associated with favorable survival in breast and bladder cancer. Currently, there is no published evidence on the role of STAT5 overexpression in bladder cancer. High STAT5a expression was associated with favorable survival in lung cancer, but there was no significant association for adjusted HRs. High STAT5a expression in skin cancer also seemed to be associated with higher survival. However, recent evidence demonstrates that STAT5 overexpression is correlated with the recurrence of melanoma [36]. In contrast, high expression in prostate cancer was associated with poor survival. However, due to unbalanced proportions between expression groups and that there was only one dataset for each cancer type, we cannot make this conclusion confidently. Further investigations into STAT5 expression in melanoma and bladder cancer patients are needed.

The relationship between high STAT5a expression and overall survival in breast and lung cancer observed here is supported by the tumor and normal tissue STAT5a expression results. Breast cancer results aligned with results from a previous study that STAT5 expression is associated with favorable overall survival and increased response to hormone therapy in ER-positive breast cancer patients [23]. In addition, previous studies indicated that STAT5 activation is correlated with favorable breast cancer-specific and disease-free survival in lymph node-negative breast cancer, and that STAT5a plays a tumor suppressive role in cancers, including breast cancer [20–22,37,38]. However, since we only investigated STAT5a expression and not activation, these studies' findings may not align precisely with our results.

Results of our analysis deviate from published findings suggesting an oncogenic role of STAT5 in breast cancer and lung cancer [5,15,16,19]. STAT5a shares more than 90% of amino acid sequence identity with STAT5b and shares a similar structure giving rise to redundant functions in cellular processes, such as cell proliferation and apoptosis [39,40]. In addition, STAT5a has overlapping functions with STAT3 protein in cancer development and progression [41]. Because of the similar functions between STAT5a, STAT5b, and STAT3, it is possible that other genes could potentially compensate for changes in the expression of one of these genes. Indeed, it has been shown that in breast and liver cancer, STAT5 signaling can offset the oncogenic effects of STAT3 [17,24]. Thus, it is also possible that specific STAT proteins have reciprocal roles, and the function of STAT5a depends on different circumstances, which could explain the contradictory findings. Future studies examining the expression and functions of STAT5 and STAT3 proteins in various cancers may sort out the relationships between STAT proteins.

A possible mechanistic explanation for the association of higher STAT5a with favorable overall survival in breast cancer might be the role of STAT5a in promoting heterochromatin formation [25]. As a non-canonical function, unphosphorylated STAT proteins (uSTATs), including uSTAT5 and uSTAT3, can associate with Heterochromatin Protein 1 (HP1) to stabilize heterochromatin formation, resulting in tumor suppression [25,42,43]. Indeed, it has been shown that uSTAT5A functions strikingly similar to HP1α in gene repression, and that many of the genes repressed by uSTAT5A and HP1α in common are overexpressed in colon cancer cells, and that uSTAT5a overexpression suppresses tumor growth [25]. In addition, genomic studies have shown that uSTAT5 is mainly involved in gene repression; that activation of the JAK/STAT pathway causes genome-wide redistribution of chromatin-bound STAT5 to traditional STAT targets, due to conversion of uSTAT5 to pSTAT5, and that either STAT5 activation or its depletion causes derepression of differentiation genes [44]. These findings are consistent with our analyses implicating STAT5A overexpression in breast cancer suppression.

This study has several limitations. First, not all clinicopathological factors could be included in the multivariate cox regression analysis, and individual-level clinical data were not available for all datasets. Missing covariate data may introduce biased adjusted HR estimates. Second, the statistical approach to determine cut-off values to distinguish high and low expression levels of STAT5a may not reflect the biology of the protein. In addition, methods of measuring the expression of a gene, such as an array type, probe selection, preparation of tumor samples, and possible experimental errors, may introduce information bias, influencing gene expression measurements. Third, meta-regression analysis and subgroup analysis could not fully explain significant heterogeneity between datasets. Heterogeneity can stem from differences in patient backgrounds in each dataset, especially since some datasets consist of specific sub-populations. Finally, there was statistical evidence of publication bias.

In summary, our systematic review study shows that high STAT5a expression is associated with favorable survival in breast cancer, suggesting that STAT5a could be a potential prognostic biomarker. Since previous studies investigated the relationship between activation of STAT5a and cancer development and progression, further investigations focusing on the relationship between STAT5a expression and cancer progression, and survival are needed to understand this relationship. Future investigations looking at the effect of gene alterations of STAT5a are also needed to understand the role of STAT5a in various cancers.

## Supplementary Material

Supplementary Figure S1 and Tables S1-S6

## Data Availability

All supporting data are included within the main article and its supplementary files.

## Open Access

Open access for this article was enabled through a transformative open access agreement between Portland Press and the University of California.

## Abbreviations

BLCA: bladder urothelial carcinoma
BRCA: breast invasive carcinoma
CI: cinfidence interval
COAD: colon adenocarcinoma
DLBC: lymphoid neoplasm diffuse large B-cell lymphoma
GBM: glioblastoma multiforme
HP1: Heterochromatin Protein 1
HR: hazard ratio
LAML: acute myeloid leukemia
LGG: brain lower grade glioma
LUAD: lung adenocarcinoma
LUSC: lung squamous cell carcinoma
NSCLC: non-small cell lung cancer
OV: ovarian serous cystadenocarcinoma
PUMA: p53 up-regulated modulator of apoptosis
PRAD: prostate adenocarcinoma
READ: rectum adenocarcinoma
ROS: reactive oxygen species
SKCM: skin cutaneous melanoma
STAT5: signal transducer and activator of transcription 5
uSTAT: unphosphorylated STAT protein
