# Supplementary material for: Expression and prognostic role of STAT5a across cancer types
Source: Biosci Rep. 2023 Aug 2;43(8):BSR20230612. doi: 10.1042/BSR20230612 (PMC10407157; doi:10.1042/BSR20230612)
Supplement: Supplementary Figure S1 and Tables S1-S6 [file BSR-2023-0612_supp.pdf]

Supplementary Information

Expression and Prognostic Role of STAT5a Across Cancer Types

Christine Maninang, Jinghong Li, and Willis X. Li

Department of Medicine
University of California San Diego
La Jolla, CA, United States

[*] Corresponding author: Willis X. Li (wxli@health.ucsd.edu)

Table S1. Dataset information from Prognoscan database

Dataset	Cancer	Subtype	Cohort	Contributor	Array Type	Probe	N
GSE5287	Bladder		Aarhus (1995-2004)	Als	HG-U133A	203010_at	30
GSE13507	Bladder		Cheongju	Kim	Human-6 v2	ILMN_1753547	165
GSE12417-GPL96	Hematological	AML	AMLCG (1999–2003)	Metzeler	HG-U133A	203010_at	163
GSE12417-GPL570	Hematological	AML	AMLCG (2004)	Metzeler	HG-U133_Plus_2	203010_at	79
GSE5122	Hematological	AML	San Diego	Raponi	HG-U133A	203010_at	58
GSE8970	Hematological	AML	San Diego	Raponi	HG-U133A	203010_at	34
GSE4475	Hematological	B-cell lymphoma	Berlin (2003–2005)	Hummel	HG-U133A	203010_at	158
E-TABM-346	Hematological	DLBCL	GELA (1998–2000)	Ais	HG-U133A	203010_at	53
GSE16131-GPL96	Hematological	Follicular lymphoma	NCI (1974-2001)	Dave	HG-U133A	203010_at	180
GSE4271-GPL96	Brain	Astrocytoma	MDA	Phillips	HG-U133A	203010_at	77
GSE7696	Brain	Glioblastoma	Lausanne	Murat	HG-U133_Plus_2	203010_at	70
MGH-glioma	Brain	Glioma	CBTTB, MGH, BWH, CH	Nutt	HG-U95A	40458_at	50
GSE4412-GPL96	Brain	Glioma	UCLA (1996-2003)	Freije	HG-U133A	203010_at	74
GSE3143	Breast		Duke	Bild	HG-U95A	506_s_at	158
GSE9893	Breast		Montpellier, Bordeaux, Turin (1989-2001)	Chanrion	MLRG Human 21K V12.0	5190	155
GSE1456-GPL96	Breast		Stockholm (1994-1996)	Pawitan	HG-U133A	203010_at	159
E-TABM-158	Breast		UCSF, CPMC (1989-1997)	Chin	HG-U133A	203010_at	117
GSE7390	Breast		Uppsala, Oxford, Stockholm, IGR, GUYT, CRH (1980-1998)	Desmedt	HG-U133A	203010_at	198
GSE12945	Colorectal		Berlin	Staub	HG-U133A	203010_at	62
GSE17536	Colorectal		MCC	Smith	HG-U133_Plus_2	203010_at	177
GSE17537	Colorectal		VMC	Smith	HG-U133_Plus_2	203010_at	55
jacob-00182-CANDF	Lung	Adenocarcinoma	CAN/DF	Shedden	HG-U133A	203010_at	82
HARVARD-LC	Lung	Adenocarcinoma	Harvard	Beer	HG-U95A	506_s_at	84
jacob-00182-HLM	Lung	Adenocarcinoma	HLM	Shedden	HG-U133A	203010_at	79
MICHIGAN-LC	Lung	Adenocarcinoma	Michigan (1994-2000)	Beer	HuGeneFL	U43185_s_at	86
jacob-00182-MSK	Lung	Adenocarcinoma	MSK	Shedden	HG-U133A	203010_at	104
GSE13213	Lung	Adenocarcinoma	Nagoya (1995-1999, 2002-2004)	Tomida	G4112F	A_23_P207367	117
GSE31210	Lung	Adenocarcinoma	NCCRI	Okayama	HG-U133_Plus_2	203010_at	204
jacob-00182-UM	Lung	Adenocarcinoma	UM	Shedden	HG-U133A	203010_at	178
GSE11117	Lung	NSCLC	Basel (2002-2005)	Baty	Novachip human 34.5k	H200007733	41
GSE3141	Lung	NSCLC	Duke	Bild	HG-U133_Plus_2	203010_at	111

GSE14814	Lung	NSCLC	JRB.10	Zhu	HG-U133A	203010_at	90
GSE4573	Lung	Squamous cell carcinoma	Michigan (1991-2002)	Raponi	HG-U133A	203010_at	129
GSE17710	Lung	Squamous cell carcinoma	UNC	Wilkerson	Agilent-UNC-custom-4X44K	41246	56
GSE9891	Ovarian		AOCS, RBH, WH, NKI-AVL (1992-2006)	Tothill	HG-U133_Plus_2	203010_at	278
DUKE-OC	Ovarian		Duke	Bild	HG-U133A	203010_at	133
GSE8841	Ovarian		Milan (1992-2003)	Marchini	G4100A	12301	81
GSE26712	Ovarian		MSKCC (1990-2003)	Bonome	HG-U133_Plus_2	203010_at	185
GSE17260	Ovarian		Niigata (1997-2008)	Yoshihara	G4112A	A_23_P207367	110
GSE14764	Ovarian		TOC	Denkert	HG-U133A	203010_at	80
GSE16560	Prostate		Sweden (1977-1999)	Sboner	6K DASL	DAP4_2174	281
GSE19234	Skin	Melanoma	NYU	Bogunovic	HG-U133_Plus_2	203010_at	38

AML= acute myelocytic leukemia; DLBCL = diffuse large B-cell lymphoma; NSCLC = non-small cell lung cancer

Table S2. Covariate information of datasets used in multivariate Cox regression analysis

Dataset	Cancer	Age (Years) Range (Median)	Sex	Grade	Stage	Other Clinical Characteristics
GSE13507	Bladder	24 – 88 (66)	F: 18.2%<br>M: 81.8%	Low: 63.6%.<br>High: 36.4%		Therapy: systematic chemotherapy: 16.4%
GSE12417-GPL96	Hematological	17-83(58)				
GSE12417-GPL570	Hematological	18-85(62)				
GSE5122	Hematological	19-85(63)	F: 51.7%<br>M: 48.3%			Tipifarnib best response:<br>CR: 5.17 % PR: 1.72%<br>hematologic improvement: 10.34 % SD: 6.90%<br>NR: 75.86%
GSE8970	Hematological	63-85(73)	F: 32.4%<br>M: 67.6%			Attribute: organ dysfunction: 55.9%<br>Attribute: prior myelodysplastic syndromes (MDS): 73.5%<br>Tipifarnib response: CR: 26.5%. hematological improvement: 11.8% PR: 38.2%<br>SD: 23.5%
GSE4475	Hematological	2-93(63)	F: 43%<br>M: 57%			
E-TABM-346	Hematological	59 – 79(69)	F: 49.1%<br>M: 50.9%	2: 18.9%<br>3: 49.1%<br>4: 17.0%<br>5: 15.1%		
GSE4271-GPL96	Brain	22-82(45)	F: 32.5%<br>M: 67.5%	3: 27.3%<br>4: 72.7%		
GSE7696	Brain	26.4-70.3 (52.2)	F: 27.1%<br>M: 72.9%	_		
GSE4412-GPL96	Brain	18-82(42.5)	F: 62.2%<br>M: 37.8%	3: 32.4%<br>4: 67.6%		
GSE9893	Breast cancer	42.6 – 92.4 (68.2)				Therapy: adjuvant therapy: TAM: 21.9% X-RAY+TAM: 73.5%<br>X-RAY+TAM+LHRH: 4.5%
E-TABM-158	Breast cancer					Positive for ER marker: 63.2%<br>N score: N0: 43.6%<br>N1: 56.4%
GSE7390	Breast cancer	24 – 60(46)				Positive for ER marker: 67.7%<br>Tumor size range: 0.6 – 5.0 cm (median: 2 cm)
GSE12945	Colorectal cancer	38 – 87(65)	0: 45.2%<br>1: 54.8%	2:50%<br>3:50%	I: 21.0%<br>II: 37.1%<br>III: 33.9%<br>IV: 8.1%	
GSE17536	Colorectal cancer	26- 92(66)	F: 47.6%<br>M: 52.4%	1: 10.3%<br>2: 76.6%<br>3: 13.1%	I: 16.6%<br>II:	Ethnicity:<br>Caucasian: 85.3% Black: 5.1% Hispanic: 0.6%

					37.9%<br>III: 38.6%<br>IV: 6.9%	Other: 9.0%
GSE17537	Colorectal cancer	23- 94(62)	F: 52.7%<br>M: 47.3%		I: 7.3%<br>II: 27.3%<br>III: 34.5%<br>IV: 30.9%	
jacob-00182-CANDF	Lung cancer	35- 79 (61)	F: 43.9%<br>M: 56.1%			N score: N0: 70.7%. N1: 29.3%<br>T score: T1: 20.7% T2: 76.8% T3: 2.4%
HARVARD-LC	Lung cancer	33-88(64)	F: 57.1%<br>M: 42.9%		I: 74%<br>II: 17%<br>III: 9%	
jacob-00182-HLM	Lung cancer	36- 87(68)	F: 49.4%<br>M: 50.6%			
MICHIGAN-LC	Lung cancer	40.9-84.6(63.7)	F: 59.3%<br>M: 40.7%		I: 77.9%<br>III: 22.1%	
jacob-00182-MSK	Lung cancer	38-82(65)	F: 64.4%<br>M: 35.6%			N score: N0: 61.5% N1: 19.2% N2: 19.2%<br>T score: T1: 31.7% T2: 64.4%. T3: 3.8%
GSE13213	Lung cancer	38- 84(61)	F: 48.7%<br>M: 51.3%		I: 56%<br>II: 15%<br>III: 29%	KRAS mutation status: mutant: 2.8% wildtype: 87.2%
GSE31210	Lung cancer	30-76(61)	F: 53.4%<br>M: 46.6%		I: 79.4%<br>II: 20.6%	Marker MYC: high: 7.8%. low: 91.7%. not detected: 0.5%<br>Smoking status: ever-smoker: 48.5%. never-smoker: 51.5%<br>Gene alteration status:<br>ALK-fusion +: 3.4%<br>EGFR mutation + :56.9%<br>EGFR/KRAS/ALK -: 30.4%<br>KRAS mutation +: 9.3%
jacob-00182-UM	Lung cancer	33-86(65)	F: 43.3%<br>M: 56.7%			N score: N0: 71.9% N1: 16.3% N2: 11.8%<br>T score: T1: 49.4% T2: 39.3%. T3: 7.3% T4:3.9%
GSE11117	Lung cancer	42-80(66)	F: 34.1%<br>M: 65.9%		I: 17.1%<br>II: 19.5%<br>III: 21.9%<br>IV: 41.5%	
GSE14814	Lung cancer	38.2-81.3(63.3)	F: 25.6%<br>M: 74.4%		I: 50%<br>II: 50%	
GSE4573	Lung cancer	42-91(68)	F: 36.4%<br>M: 63.6%		I: 56%<br>II: 26%	

					III: 18%	
GSE17710	Lung cancer	41-85(67)	F: 42.9%<br>M: 57.1%	Mod: 64.3%<br>Poor: 35.7%	I: 60.7%<br>II: 34%<br>III: 5.4%	
GSE9891	Ovarian cancer	22-80(59)				
GSE8841	Ovarian cancer	21.1-87.1(49.9)		1: 16.0%<br>2: 24.7%<br>3: 40.7%<br>Borderline:18.5%	1A: 30.9%<br>1B: 6.2%<br>1C: 63%	
GSE26712	Ovarian cancer	-				Attribute: cytoreductive surgery outcome: optimal: 48.6% suboptimal: 51.4%
GSE17260	Ovarian cancer				III: 84.6%<br>IV: 15.5%	Attribute: cytoreductive surgery outcome: not optimal: 48.2% optimal: 51.8%
GSE14764	Ovarian cancer			1: 3.8%<br>2: 28.7%<br>3: 67.5%	I: 10%<br>II: 1.2%<br>III: 86.2%<br>IV: 2.5%	
GSE16560	Prostate cancer	51-91(74)				Gleason score range: 6-10<br>Median: 7
GSE19234	Skin cancer	30-92(62.5)	F: 36.8%<br>M: 63.2%		III: 86.8%<br>IV: 13.2%	

F = female, M= male, ER= estrogen receptor, KRAS= Kirsten rat sarcoma virus gene, EGFR= epidermal growth factor receptor, ALK= anaplastic lymphoma kinase, TAM= tamoxifen, LHRH= luteinizing-hormone-releasing hormone agonists

Table S3. Covariates added in multivariate Cox regression analysis

Cancer Type	Dataset	Covariates
**Bladder**	GSE13507	Age (continuous), Sex (factor), Grade (factor), Systemic chemotherapy (factor)
**Hematological**	GSE12417-GPL96	Age (continuous)
	GSE12417-GPL570	Age (continuous)
	GSE5122	Age (continuous), Sex (factor), Tipifarnib best response (factor)
	GSE8970	Age (continuous), Sex (factor), Organ Dysfunction (factor), Prior MDS (factor), Tipifarnib response (factor)
	GSE4475	Age (continuous), Sex (factor)
	E-TABM-346	Age (continuous), Sex (factor), Grade (continuous)
**Brain**	GSE4271-GPL96	Age (continuous), Sex (factor), Grade (continuous)
	GSE7696	Age (continuous), Sex (factor)
	GSE4412-GPL96	Age (continuous), Sex(factor), Grade (continuous)
**Breast**	GSE9893	Age (continuous), Adjuvant therapy (factor)
	E-TABM-158	ER marker (factor), N score (factor)
	GSE7390	Age (continuous), ER marker (factor), Tumor size (continuous)
**Colorectal**	GSE12945	Age (continuous), Sex (factor), Grade (continuous), Stage (factor)
	GSE17536	Age (continuous), Sex (factor), Grade (continuous), Stage (factor), Ethnicity (factor)
	GSE17537	Age (continuous), Sex (factor), Stage (factor)
**Lung**	jacob-00182-CANDF	Age (continuous), Sex (factor), N score (factor), T score (factor)
	HARVARD-LC	Age (continuous), Sex (factor), Stage (factor)
	jacob-00182-HLM	Age (continuous), Sex (factor)
	MICHIGAN-LC	Age (continuous), Sex (factor), Stage (factor)
	jacob-00182-MSK	Age (continuous), Sex (factor), N score (factor), T score (factor)
	GSE13213	Age (continuous), Sex (factor), Stage (factor), KRAS mutation (factor), TP53 mutation (factor)
	GSE31210	Age (continuous), Sex (factor), Stage (factor), Smoking Status (factor), Gene alteration status (factor), MYC marker (factor)
	jacob-00182-UM	Age (continuous), Sex (factor), N score (factor), T score (factor)
	GSE11117	Age (continuous), Sex (factor), Stage (factor)
	GSE14814	Age (continuous), Sex (factor), Stage (factor)
	GSE4573	Age (continuous), Sex (factor), Stage (factor)
	GSE17710	Age (continuous), Sex (factor), Grade (factor), Stage (factor)
**Ovarian**	GSE9891	Age (continuous)
	GSE8841	Age (continuous), Grade (factor), Cancer substage (factor)
	GSE26712	Cytoreductive surgery outcome (factor)
	GSE17260	Stage (factor), Cytoreductive surgery outcome (factor)
	GSE14764	Grade (continuous), Stage (factor)
**Prostate**	GSE16560	Age (continuous), Gleason score (continuous)
**Skin**	GSE19234	Age (continuous), Sex (factor), Stage (factor)

Table S4. The association of high STAT5a expression and overall survival in cancer patients

Cancer Type	Dataset	N	Unadjusted lnHR (95% CI)	Cox-p value (Univariate)	Adjusted lnHR (95% CI)	Cox p-value (multivariate)
**Bladder**	GSE5287	30	-1.725 (-2.905, -0.5452)	0.00416		
	GSE13507	165	-0.6424 (-1.249, -0.0354)	0.038	-0.5452 (-1.173, 0.08342)	0.089
**Hematological**	GSE12417-GPL96	163	0.7673 (0.3478, 1.187)	0.000339	0.8436 (0.4207, 1.266)	9.23E-05
	GSE12417-GPL570	79	0.8764 (0.2897, 1.463)	0.0034	0.7783 (0.1840, 1.372)	0.0102
	GSE5122	58	0.7099 (0.07973, 1.339)	0.0271	0.5991 (-0.1421, 1.340)	0.1132
	GSE8970	34	-0.4274 (-1.302, 0.4472)	0.338	-0.8650 (-2.078, 0.3478)	0.162
	GSE4475	158	0.277 (-0.5021, 1.056)	0.486	-0.3011 (-1.103, 0.5014)	0.462
	E-TABM-346	53	-0.4099 (-1.134, 0.3141)	0.267	-0.7492 (-1.543, 0.04497)	0.0644
	GSE16131-GPL96	180	0.3865 (-0.2458, 1.019)	0.231		
**Brain**	GSE4271-GPL96	77	0.6887 (0.07046, 1.306)	0.0289	0.4516 (-0.1791, 1.082)	0.1605
	GSE7696	70	-0.9594 (-1.979, 0.06015)	0.0652	-0.8598 (-1.894, 0.1748)	0.1034
	MGH-glioma	50	-0.8545 (-1.563, -0.1462)	0.018		
	GSE4412-GPL96	74	0.292 (-0.3260, 0.9099)	0.354	0.07562 (-0.5616, 0.7130)	0.81604
**Breast**	GSE3143	158	-0.7939 (-1.376, -0.2141)	0.00728		
	GSE9893	155	-0.673 (-1.328, -0.01816)	0.044	-0.6012 (-1.256, 0.05354)	0.0721
	GSE1456-GPL96	159	-2.154 (-4.139, -0.1688)	0.0335		
	E-TABM-158	117	-0.4852 (-1.202, 0.2319)	0.185	-0.3988 (-1.123, 0.3250)	0.28
	GSE7390	198	-2.490 (-4.468, -0.5118)	0.0136	-2.265 (-4.252, -0.2781)	0.0255
**Colorectal**	GSE12945	62	-1.737 (-2.893, -0.5805)	0.00324	-1.630 (-3.143, -0.1173)	0.0347
	GSE17536	177	-0.2428 (-0.7444, 0.2585)	0.343	-0.3440 (-0.8675, 0.1794)	0.1977
	GSE17537	55	1.211 (0.1115, 2.311)	0.0308	1.124 (0.001399, 2.247)	0.0497
**Lung**	jacob-00182-CANDF	82	-0.4515 (-1.151, 0.2476)	0.206	-0.1389 (-0.8761, 0.5983)	0.71193
	HARVARD-LC	84	-0.6622 (-1.301, -0.02358)	0.0421	-0.6808 (-1.382, 0.02069)	0.057154
	jacob-00182-HLM	79	0.2669 (-0.2735, 0.8074)	0.333	0.06735 (-0.5285, 0.6632)	0.825
	MICHIGAN-LC	86	-1.105 (-2.096, -0.1134)	0.0289	-0.6944 (-1.759, 0.3701)	0.2011
	jacob-00182-MSK	104	0.6978 (0.04688, 1.348)	0.0355	0.6949 (0.02703, 1.363)	0.04143
	GSE13213	117	-0.8805 (-1.579, -0.1822)	0.0135	-0.6164 (-1.359, 0.1258)	0.10366
	GSE31210	204	-0.3718 (-1.183 0.4395)	0.369	-0.4054 (-1.304, 0.4898)	0.374826
	jacob-00182-UM	178	0.315 (-0.2210, 0.8510)	0.249	0.7182 (0.1207, 1.316	0.01847
	GSE11117	41	0.593 (-0.4887, 1.675)	0.283	0.01471 (-1.206, 1.235)	0.9812
	GSE3141	111	-0.843 (-1.449, -0.2370)	0.0064		
	GSE14814	90	-0.4321 (-1.155, 0.2912)	0.241	-0.5006 (-1.232, 0.2311)	0.18
	GSE4573	129	-0.5231 (-1.018, -0.02819)	0.0383	-0.4907 (-1.019, 0.03730)	0.0684
	GSE17710	56	-0.9255 (-1.671, -0.1801)	0.0149	-0.7921 (-1.652, 0.06766)	0.0708
**Ovarian**	GSE9891	278	0.4797 (0.05543, 0.9042)	0.0267	0.4741(0.04879, 0.8993)	0.0288
	DUKE-OC	133	-0.2011 (-0.6882,	0.418		

			0.2859)			
	GSE8841	81	-1.324 (-2.622, -0.02552)	0.0457	-0.7985 (-2.245, 0.6476)	0.2791
	GSE26712	185	0.3574 (-0.06283, 0.7775)	0.0955	0.4276 (0.004988, 0.8502)	0.04735
	GSE17260	110	0.6203 (-0.08219, 1.323)	0.0835	0.7245 (0.01094, 1.438)	0.0466
	GSE14764	80	-0.7326 (-1.644, 0.1781)	0.115	-0.6702 (-1.622, 0.2814)	0.168
**Prostate**	GSE16560	281	0.6987 (0.1553, 1.242)	0.0117	0.736 (0.1906, 1.281)	0.00818
**Skin**	GSE19234	38	-1.788 (-2.975, -0.6004)	0.00317	-1.144 (-2.615, 0.3264)	0.127

Table S5. Amplification of STAT5a data across cancer types

STAT5A (chr17:37693090-37717484)

Cancer Subset	In Peak?	Nearest Peak (click link to launch IGV)	#Genes in Peak	Q-value	Frequency of Amplification		
					Overall	Focal	High-level
all_cancers	No	chr17:35087126-35236409	6	1.0	0.2293	0.06	0.0093
all_neural	Yes	chr17:11922171-42715679	463	1.0	0.4009	0.0507	0.0553
Colorectal	No	chr17:34938927-35519475	19	0.381	0.2671	0.1615	0.0
Esophageal adenocarcinoma	No	chr17:36989609-37319012	13	0.425	0.3288	0.1507	0.0137
Lung NSC	No	chr17:34950738-35236409	9	0.787	0.3697	0.0941	0.0095
Hepatocellular	No	chr17:19117656-19426327	7	0.9	0.2893	0.0992	0.0083
Renal	No	chr17:40699630-41015664	3	0.922	0.2143	0.1032	0.0
all_lung	No	chr17:34950738-35236409	9	0.961	0.3747	0.0943	0.009
Esophageal squamous	No	chr17:34957266-35272329	10	1.0	0.2955	0.0682	0.0
Lung SC	No	No peak on chromosome	0	1.0	0.45	0.075	0.0
Myeloproliferative disorder	No	No peak on chromosome	0	1.0	0.0	0.0	0.0
Ovarian	No	chr17:47316963-78605474	332	1.0	0.1553	0.0194	0.0097
Acute lymphoblastic leukemia	No	No peak on chromosome	0	1.0	0.0793	0.0	0.0
Breast	No	chr17:35087126-35272329	7	1.0	0.1605	0.0494	0.0123
Dedifferentiated liposarcoma	No	No peak on chromosome	0	1.0	0.1132	0.0377	0.0
Glioma	No	No peak on chromosome	0	1.0	0.1463	0.0488	0.0
Medulloblastoma	No	chr17:53715836-56717580	34	1.0	0.4531	0.0469	0.0547
Melanoma	No	chr17:41471733-78605474	429	1.0	0.2432	0.018	0.0
Prostate	No	No peak on chromosome	0	1.0	0.1304	0.0217	0.0
all_epithelial	No	chr17:35087126-35236409	6	1.0	0.28	0.0833	0.0072
all_hematologic	No	No peak on chromosome	0	1.0	0.0572	0.0	0.0014
all_sarcoma	No	chr17:17562861-21703267	47	1.0	0.1736	0.0556	0.0069

Tumorscape database analysis of STAT5a amplification in different cancer types

Table S6. Deletion of STAT5a data across cancer types

STAT5A (chr17:37693090-37717484)

Cancer Subset	In Peak?	Nearest Peak (click link to launch IGV)	#Genes in Peak	Q-value	Frequency of Deletion		
					Overall	Focal	High-level
all_cancers	Yes	chr17:37319300-37970046	22	1.56E-6	0.123	0.0425	6.0E-4
all_epithelial	Yes	chr17:37319300-37970046	22	3.17E-5	0.1605	0.0502	0.001
Breast	Yes	chr17:35837132-39658433	137	1.43E-4	0.4362	0.1358	0.0041
Ovarian	Yes	chr17:25917773-38819347	249	0.347	0.3689	0.068	0.0
Dedifferentiated liposarcoma	Yes	chr17:23631847-39070759	306	0.621	0.2075	0.0943	0.0
Melanoma	Yes	chr17:24384534-51175244	429	0.837	0.1802	0.0631	0.0
Colorectal	Yes	chr17:6888-78605474	1115	1.0	0.0994	0.0062	0.0
Lung SC	Yes	chr17:6738539-42166282	565	1.0	0.15	0.1	0.0
Myeloproliferative disorder	Yes	chr17:6888-78605474	1115	1.0	0.0047	0.0	0.0
all_sarcoma	No	chr17:24099550-31217201	85	0.0169	0.1806	0.0868	0.0
Prostate	No	chr17:39354620-40542083	33	0.202	0.1087	0.087	0.0109
all_neural	No	No peak on chromosome	0	0.367	0.0553	0.0323	0.0
all_lung	No	chr17:26185629-26896751	9	0.412	0.084	0.0336	0.0
Lung NSC	No	chr17:21100623-33308219	157	0.532	0.0805	0.03	0.0
Medulloblastoma	No	No peak on chromosome	0	0.597	0.0312	0.0234	0.0
Glioma	No	No peak on chromosome	0	0.937	0.1707	0.0732	0.0
Acute lymphoblastic leukemia	No	chr17:25917773-27283563	14	1.0	0.0179	0.0051	0.0
Esophageal adenocarcinoma	No	chr17:75915141-77213225	19	1.0	0.1781	0.0411	0.0
Esophageal squamous	No	No peak on chromosome	0	1.0	0.2273	0.0227	0.0
Hepatocellular	No	No peak on chromosome	0	1.0	0.0661	0.0165	0.0
Renal	No	No peak on chromosome	0	1.0	0.0317	0.0159	0.0
all_hematologic	No	chr17:25917773-27368624	16	1.0	0.0157	0.0057	0.0

Tumorscape database analysis of STAT5a deletions in different cancer types. Cancer types highlighted in orange indicates that STAT5a is significantly focally amplified or deleted. Q value threshold is set at 0.25.

Figure S1. Gene alterations of STAT5a in cancer types

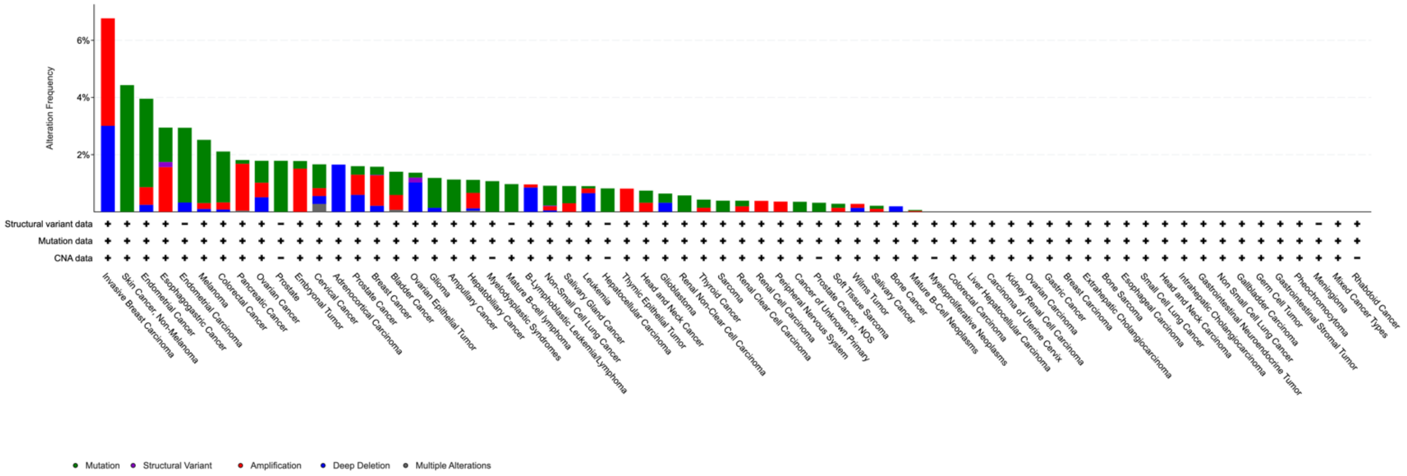

Figure S1. Alteration frequency and gene alteration type of STAT5a was examined using cBioPortal. Only cancer types with at least 100 samples are shown.
